# Genome-wide analysis of the soybean root transcriptome reveals the impact of nitrate on alternative splicing

**DOI:** 10.1093/g3journal/jkab162

**Published:** 2021-05-10

**Authors:** Binhui Guo, Yi Dai, Lin Chen, Zhenzhi Pan, Li Song

**Affiliations:** 1 Joint International Research Laboratory of Agriculture and Agri-Product Safety, the Ministry of Education of China, Jiangsu Key Laboratory of Crop Genomics and Molecular Breeding, Yangzhou University, Yangzhou, Jiangsu 225009, China; 2 Basic Experimental Teaching Center of Life Science, Yangzhou University, Yangzhou, Jiangsu 225009, China

**Keywords:** soybean, alternative splicing, nitrate, transcriptome, root development

## Abstract

In plants, nitrate acts not only as a signaling molecule that affects plant development but also as a nutrient. The development of plant roots, which directly absorb nutrients, is greatly affected by nitrate supply. Alternative gene splicing plays a crucial role in the plant stress response by increasing transcriptome diversity. The effects of nitrate supply on alternative splicing (AS), however, have not been investigated in soybean roots. We used high-quality high-throughput RNA-sequencing data to investigate genome-wide AS events in soybean roots in response to various levels of nitrate supply. In total, we identified 355 nitrate-responsive AS events between optimal and high nitrate levels (NH), 335 nitrate-responsive AS events between optimal and low nitrate levels (NL), and 588 nitrate-responsive AS events between low and high nitrate levels (NLH). RI and A3SS were the most common AS types; in particular, they accounted for 67% of all AS events under all conditions. This increased complex and diversity of AS events regulation might be associated with the soybean response to nitrate. Functional ontology enrichment analysis suggested that the differentially splicing genes were associated with several pathways, including spliceosome, base excision repair, mRNA surveillance pathway and so on. Finally, we validated several AS events using reverse transcription–polymerase chain reaction to confirm our RNA-seq results. In summary, we characterized the features and patterns of genome-wide AS in the soybean root exposed to different nitrate levels, and our results revealed that AS is an important mechanism of nitrate-response regulation in the soybean root.

## Introduction

Nitrogen (N) is an essential macronutrient for plant growth and crop production (Lawlor *et al.* 2001). Among the different forms of N, nitrate (${\text{NO}}_{3}^{-}$) is the main source of nutrients in most agricultural soils, as well as for most cereals (Tegeder and Masclaux-Daubresse 2018). Root development and root system architecture are closely related to nitrate supply (Shahzad and Amtmann 2017). Low or moderate nitrate availability promotes root growth and increases the root–shoot ratio, thereby enhancing the ability of the root system to acquire nutrients (Gruber *et al.* 2013; Tian *et al.* 2014). Nitrate supply may induce root hair development after a period of N-starvation (Canales *et al.* 2017). When nitrate supply is very low or very high, however, lateral root branching and elongation are inhibited (Sun *et al.* 2017). In addition, nitrate acts as an important signaling molecule in many biology processes, including nitrogen transport, nitrogen assimilation, and lateral root development (Remans *et al.* 2006; Wang *et al.* 2012; Medici and Krouk 2014).

Forms of alternative splicing (AS) include intron retention (IR), skipped exon (SE), alternative 5′ splicing site (A5SS), alternative 3′ splicing site (A3SS), and mutually exclusive exon (MXE; Syed *et al.* 2012). Environmental conditions may cause different segments of the original RNA to be omitted from the mRNA, resulting in the translation of the alternate mRNA sequence into an entirely different protein that may have an altered structure, function, or subcellular location (Nilsen and Graveley 2010; Kriechbaumer *et al.* 2012; Staiger and Brown 2013; Gupta *et al.* 2017). Thus, AS plays a vital regulatory role at the posttranscriptional level in response to environmental conditions, contributing to transcriptomic plasticity in plants (Syed *et al.* 2012; Reddy *et al.* 2013). In addition, the AS regulation pattern of genes is related to nutrients in environment. For example, changes in AS patterns were nutrient specific in *Arabidopsis* (Li *et al.* 2013; Nishida *et al.* 2017). AS also plays a key role in regulating mineral nutrient homeostasis in rice, and several serine/arginine-rich proteins act as critical regulators of nutrition (Dong *et al.* 2018). Both AS isoforms of the nutrition response and root growth gene (NRRa and NRRb) played negative regulatory roles in rice root growth, depending on macronutrient availability (Zhang et al. 2012). Differences in the relative abundances of AS variants of the rice sulfate transporter gene (*OsSultr1; 1*) were correlated with sulfur levels in the growth medium (Kumar *et al.* 2011).

Several recent studies have explored the interactions among AS, nitrate, and root development (Fang *et al.* 2017; Ishizawa *et al.* 2019; Wang *et al.* 2020). It has been reported that more than 1000 maize genes have exhibited specific AS modulations in response to N treatment, which altered early N responses (Wang *et al.* 2020). AS variants of rice amino acid transporter genes (*OsAAT*s) were regulated by natural variations in expression ratios in rice grown with various levels of nitrogen; these AS variants then regulated nutrient uptake and allocation (Fang *et al.* 2017). Root hairs play important roles in water absorption and nutrient uptake, and splicing factor 3b is involved in pre-mRNA splicing, which is associated with root hair development in response to light signals (Ishizawa *et al.* 2019). Thus, the regulation of pre-mRNA splicing is necessary both for plant responses to environmental change and for root development (Braunschweig *et al.* 2013). Wei *et al.* (2017, 2020) found that AS increased the complexity of gene expression associated with drought adaptation in deep-rooted rice, but that gene expression levels did not change significantly. Therefore, an improved understanding of transcript isoform diversity might help to clarify the relationship between nitrate and root development.

The soybean is one of the most economically important legumes, providing vegetable protein for people of the world (2019 Soystats). Both N_2_ fixation from root nodules and inorganic nitrogen assimilation from roots are important mechanisms by which to increase yield without requiring overfertilization, which results in severe environmental pollution (Xu et al. 2012). However, root nodules are not yet established during the early growth of the soybean, and root growth is significantly affected by the availability of environmental nutrients, especially nitrate (Saito *et al.* 2014). In addition, global genetic analysis has suggested that more than 63% of all multiexonic soybean genes underwent AS, and showed that more AS events occurred during earlier developmental stages than the older developmental stages for the same type of tissue (Shen *et al.* 2014). Furthermore, variations in soybean gene structure and transcriptional levels both affected the AS regulation (Shen *et al.* 2014). The relationship between AS frequency and nitrate levels; however, has not yet been explored in soybeans.

We analyzed RNA-seq data collected from soybeans grown with optimal nitrate supply (NN), low nitrate supply (NL), and high nitrate supply (NH) to investigate the effects of nitrate on AS. Our results showed that the relative frequencies of 859 gene isoforms differed significantly among plants grown at different nitrate concentrations. These findings will help to accelerate gene identification and to clarify the role of AS in nitrate uptake during early soybean growth.

## Materials and methods

### Plant materials and nitrate treatments

Seeds of soybean variety Williams 82 (*Glycine max* L.) were sterilized using chlorine gas following the protocols of Paz *et al.* (2006). Sterilized seeds were cultivated in Murashige and Skoog (MS) liquid media supplemented with 18.81 mM KNO_3_ (optimal, group NN), 56.43 mM (high nitrate, group HN), or 6.27 mM KNO_3_ (low nitrate, group LN). Seeds were grown in a growth chamber at 25°C for 4 weeks, with a 16/8 h (light/dark) photoperiod and 60% humidity. We did not add any NH_4_NO_3_ to any treatments to exclude the effects of ${\text{NH}}_{4}^{-}$. KCl was used to make up the concentration of K across different treatments. Each treatment was represented by three biological replicates; all replicates were run in parallel. The MS solution was changed every 5 days to maintain constant nutrient levels. Roots from each treatment were collected separately and immediately frozen in liquid nitrogen.

### RNA-seq and reference-based transcriptome assembly

We extracted total RNA using the Trizol reagent kit (Invitrogen, Carlsbad, CA, USA), following the manufacturer’s protocols. We assessed RNA quality using an Agilent 2100 Bioanalyzer (Agilent Technologies, Palo Alto, CA, USA) and checked quality using RNase-free agarose gel electrophoresis. mRNA was enriched using Oligo (dT) beads, and the enriched mRNA was used as a template for cDNA synthesis. cDNA was sequenced using an Illumina HiSeq2500 (Illumina, San Diego, CA, USA) by Gene Denovo Biotechnology Co. (Guangzhou, China).

To obtain high-quality clean reads, we used fastp (version 0.18.0; Chen *et al.* 2018) to remove adapters and low-quality bases. We used the short-reads alignment tool Bowtie2 (version 2.2.8; Langmead and Salzberg 2012) to map reads to the ribosome RNA (rRNA) database. The rRNA-mapped reads were removed. High-quality paired-end clean reads from each sample were aligned to the soybean reference genome (*G. max Wm82.a2.v1*) using HISAT2.2.4 (Kim *et al.* 2015), with “-rna-strandness RF” and other parameters set to default. We assembled mapped reads for each sample using StringTie (v1.3.1; Pertea *et al.* 2015, 2016) based on the reference genome (*G. max Wm82.a2.v1*). We calculated the fragment per kilobase of transcript per million mapped reads (FPKM) value for each transcription region value using StringTie to quantify variations in expression abundance.

### AS detection

We used rMATs (v4.0.1; Shen *et al.* 2014; http://rnaseq-mats.sourceforge.net/index.html) to identify AS events and to identify differences in AS events among samples. To remove the AS events potentially predicted by mapping error, only junctions supported by at least five uniquely mapped reads in at least one sample were considered in further analysis. The PSI change between two conditions was calculated as Inclevel differences = IncLevel1 − IncLevel2. IncLevel = (IJC_SAMPLE/IncFormLen)/[(SJC_SAMPLE/SkipFormLen) + (IJC_SAMPLE/IncFormLen)]. AS events with false discovery rates (FDRs) < 0.1 and |Δ Percent spliced in (PSI)| > 0.05 were considered to have a significant relationship with nitrate exposure. We identified five types of AS events: SE, skipped exon; MXE, mutually exclusive exon; A5SS, alternative 5′ splice site; A3SS, alternative 3′ splice site; and RI, retained intron.

### Identification of differentially expressed genes

We identified differences in gene expression based on the RNA data using DESeq2 between pairs of groups and using edgeR between pairs of samples (Robinson *et al.* 2010; Love *et al.* 2014). Genes and transcripts with FDR < 0.05 and |fold change| ≥ 1 were considered significantly differentially expressed.

### Functional annotation and gene ontology

We mapped all differentially spliced genes (DSGs) to gene ontology (GO) terms in the GO database (http://www.geneontology.org/) and calculated the numbers of genes mapped to each term. We identified the GO terms significantly enriched in the DSGs compared with the genome background using the hypergeometric test.

### Quantitative and reverse transcription–polymerase chain reactions

We reverse transcribed total RNA, pretreated with DNase I, using HiScript^®^ III RT SuperMix (Cat no. R323-01, Vazyme, Nanjing, Jiangsu, China), following the manufacturer’s instructions. To validate AS events, we performed reverse transcription–polymerase chain reactions (RT-PCRs) in 20 μl reaction volumes. Primer pairs for each gene were designed for both ends of each splice to amplify both splice variants (isoforms 1 and 2) in a single reaction. The primers used for qPCR are listed in Supplementary Table S5.

### Data availability

The data underlying this article are available in the NCBI Sequence Read Archive (SRA), with BioProject number PRJNA668854. Supplementary material is available at figshare: https://doi.org/10.25387/g3.14551446.

## Results

### Overview of the high-quality transcriptome, showing the response of the soybean root to nitrate

To investigate the regulation of AS patterns during soybean root development under high or low levels of nitrate supply, we performed high-throughput RNA-seq analyses using Illumina Highseq 2500 sequencing technology. We compared gene expression patterns among three treatments: NN (optimal nitrate supply), NL (low nitrate supply), and NH (high nitrate supply). We observed clear transcriptome differences among samples treated with different concentrations of nitrate (Supplementary Figure S1A), suggesting that nitrate content strongly affected patterns of gene expression. The sequencing coverage results indicated that there was no significant sequencing bias (Supplementary Figure S1B). The FPKM values for the samples are shown in Supplementary Figure S1C. The mapping analysis showed that about 91% of all input reads were uniquely mapped to the *G. max* reference genome, and more than 95% of all mapped reads were aligned to the exon region. Thus, our results indicated the sequencing data and transcriptome assembly obtained here were high quality and that our RNA-seq results were suitable for the identification of AS events.

Genes were identified as significantly differentially expressed if the FDR was <0.05 and the |log2| ratio was ≥1. We found that nitrate levels had a significant effect on gene expression patterns. That is, 609 genes were upregulated and 1045 genes were downregulated in the low-nitrate treatment compared with the optimal-nitrate treatment (NL), whereas 962 genes were upregulated and 321 genes were downregulated in the high-nitrate treatment compared with the optimal-nitrate treatment (NH). In addition, 1380 genes were upregulated and 703 genes were downregulated in the low-nitrate treatment compared with the NLH (Supplementary Figure S2A). These results indicated that differences in nitrate supply significantly altered gene expression patterns. Venn diagram analysis suggested that very few genes (<5%) were differentially expressed in both the NL and the NH groups (Supplementary Figure S2, B and C).

### Identification of AS events in soybean roots

**Figure 1:**
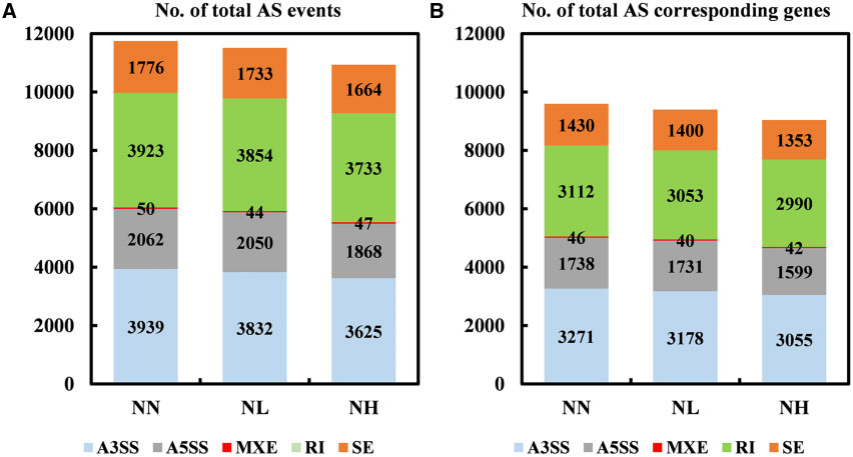
Identification of AS events in the soybean root transcriptomes in response to different nitrate levels. (A) The number of each type of AS events and (B) the number of AS corresponding genes. SE, skipped exon; MXE, mutually exclusive exon; A5SS, alternative 5' splice site; A3SS, alternative 3′ splice site; RI, retained intron.

We identified and quantified AS events based on junction counts only method using rMATs. Only junctions supported by at least five uniquely mapped reads in at least one sample were considered in further analysis. All five AS types were identified across all samples and treatments (Figure 1A). Totally, 34,200 AS events were identified from all samples. Among the five types of AS, RI, and A3SS events were the most common, followed by A5SS and SE events. Unsurprisingly, MXE events were the least common. Notably, we did not observe any significant differences in the distributions and numbers of AS event types among the three treatments. We further counted the total number of genes that corresponding to at least one AS event. The results indicated that only 28,038 genes were identified in all samples, which suggesting extensive genes undergo more than one AS event (Figure 1B). In addition, the number of AS events was identified using ASTALAVISTA program to compare the accuracy of rMATs software. As shown in Supplementary Figure S3, totally 21,391 AS events were identified from all samples. In which, RI events represented 55% of the total events and was the most abundant AS type.

### Identification of nitrate-responsive AS events in soybean root tissues

**Figure 2:**
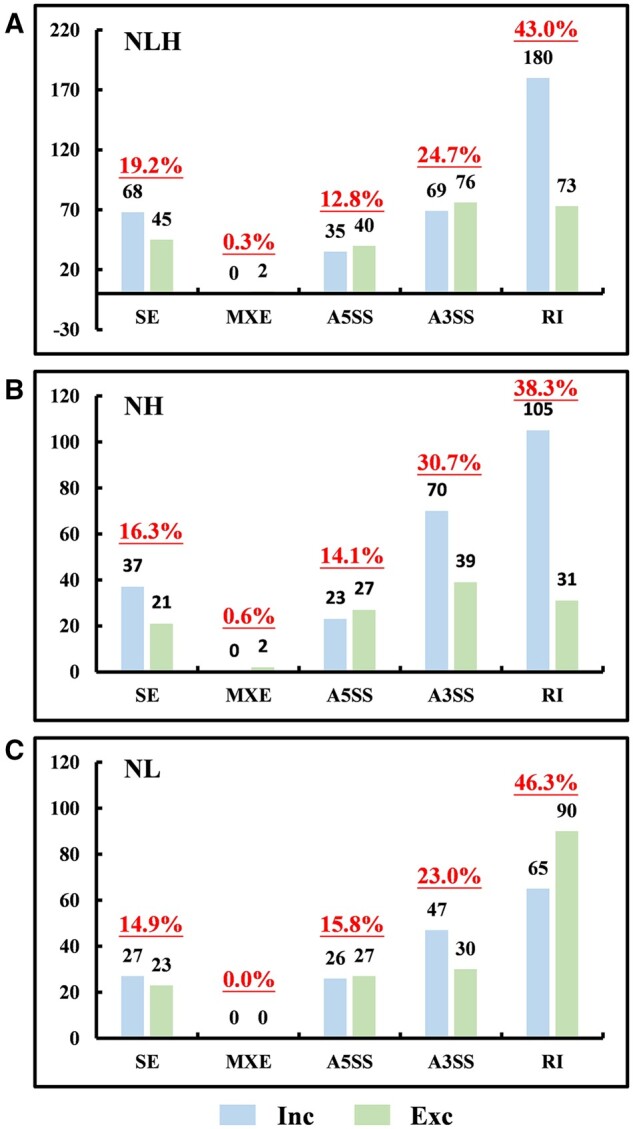
The number and relative frequencies of DAS events under different nitrate treatments. (A) Under high-nitrate conditions compared with low-nitrate conditions (NLH). (B) Under high-nitrate conditions compared with optimal nitrate conditions (NH). (C) Under low-nitrate conditions compared with optimal-nitrate conditions (NL). Exclusion events shown in green; inclusion events shown in blue. The numbers of AS events are shown along the y-axis; the types of AS events are shown along the x-axis.

To explore the AS events that might affect soybean root development in response to different levels of nitrate, we identified and quantified alternatively spliced isoforms and differential AS (DAS) events. We considered junction reads when the FDR < 0.1 and |ΔPSI| > 0.05 was nitrate-responsive AS events. We identified 355, 335, and 588 nitrate-responsive AS events under NH, NL, and NLH conditions, respectively. Of the five AS types, RI was the most abundant under NLH (43%), NH (38.3%), and NL (46.3%) conditions (Figure 2). More RI events were identified under NL conditions than under NH conditions, but more A3SS events were identified under NH conditions than under NL conditions. In addition, higher nitrate concentrations increased the number of RI-type exon inclusion events, and the lower nitrate concentrations decreased the number of RI-type exon inclusion events. Moreover, of the A3SS events identified under NH conditions, alternative exon inclusion events were much more common than exon exclusion events (Figure 2). These results suggested that nitrate concentration affected the relative frequency of different types of AS events as well as the proportions of different isoforms.

**Figure 3:**
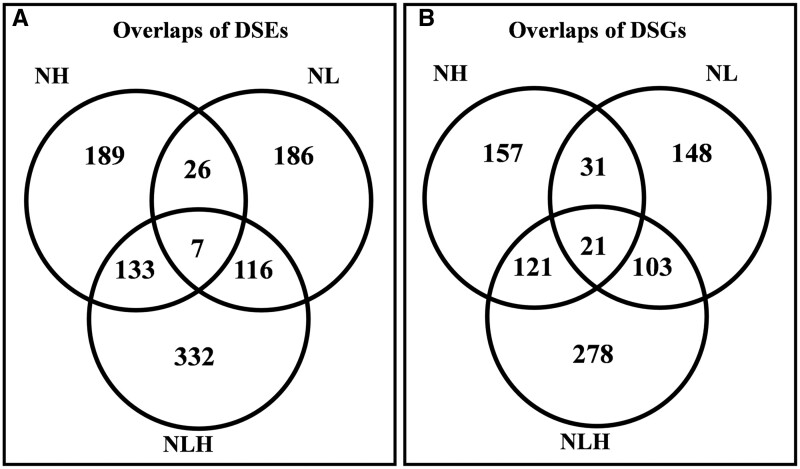
Venn diagrams showing (A) significant nitrate-responsive AS events (DSEs) and (B) genes correlated with the DSEs (DSGs) across the three different nitrate conditions.

We compared nitrate-responsive AS events among the three treatments. We identified 189 AS events unique to NH, 186 unique to NL, and 332 unique to NLH (Figure 3A). Furthermore, the types of AS events driven by nitrate differed among treatments: only 33 AS events overlapped between the NL and NH groups (Supplementary Figure S4, A–E). Genes in which the AS event differed significantly in response to stress were considered DSGs. We identified a total of 859 DSGs in the soybean root (330 under NH treatment, 303 under NL treatment, and 523 under NLH treatment). Moreover, we compared the overlap in DSGs under various nitrate conditions, and the results showed that only 52 genes were shared between the NL and NH treatments (Figure 3B). This result indicated that the regulation of nitrate-responsive AS events was closely correlated with nitrate concentration. In addition, 35 genes were common in both differentially expressed genes and DSGs (Supplementary Figure S4F). That is, these genes were both significantly differentially expressed and significantly differentially spliced in response to nitrate. These results suggested that the regulation of both gene expression level and AS in the DSGs played crucial roles in the response of the soybean root to nitrate treatment.

### Functional enrichment analysis of DSGs among nitrate concentrations

**Figure 4:**
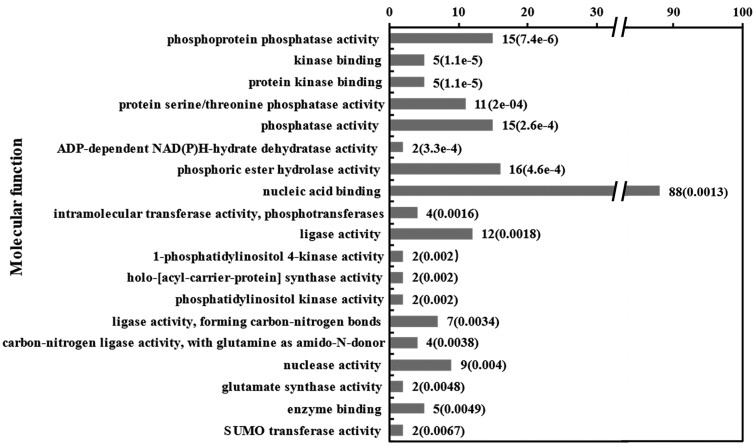
Enrichment analyses of molecular function GO terms of DSGs. GO terms (*P* < 0.01) associated with the significantly nitrate-responsive AS genes. GO subcategories are shown along the x-axis, and the number of genes associated with each subcategory is shown along the y-axis.

To investigate the functions of genes that differentially alternatively spliced in response to different levels of nitrate, we analyzed the GO enrichment (biological process, molecular function, and cellular component) of the DSGs. We found that 57 biological processes, 10 cellular components, and 19 molecular functions were significantly enriched in those DSGs (Supplementary Table S1, *P*-value < 0.01). Metabolic process and cellular process were the most enriched biological process terms (Supplementary Table S1). The majority of the top molecular function GO terms had correlations with binding and catalytic activity, such as nucleic acid binding, enzyme binding, phosphatase activity, and ligase activity and so on (Figure 4). These results indicated that different levels of nitrate triggered AS events in the genes involved in binding and metabolism. In addition, several genes associated with signaling terms were alternatively spliced, regardless of whether these genes are significantly regulated at the gene expression level at the same time. The disruption of the gene coding frame due to splicing led to the premature termination of protein translation, which seriously affected gene function (Table 1).

**Table 1:** Alternatively spliced genes associated with signaling-related GO terms

Gene name	Gene ID	AS type	Gene annotation	Effect of AS on protein	NLH log2 (fc)	NH log2 (fc)	NL log2 (fc)	NLH IncLevel difference	NH IncLevel difference	NL IncLevel difference
*GID1C*	*Glyma.20G230600*	RI	Gibberellin receptor GID1C-like	3′UTR, Not affected	−0.370	−0.269	—	0.218	—	—
*RGS1*	*Glyma.11G245800*	RI	Regulator of G-protein signaling 1-like isoform X1	Induce a shorted protein	—	—	—	0.323	0.277	—
*SRK2A*	*Glyma.06G160100*	RI	Serine/threonine-protein kinase SRK2A-like	Induce a PTC	—	—	—	0.121	—	—
*TGA4*	*Glyma.18G020900*	RI	Transcription factor TGA1	5′UTR, not affected	−0.633	−0.410	—	0.113	—	—
*NPF6.3*	*Glyma.11G031500*	A3SS	Protein NRT1/PTR FAMILY 6.3	Not affected?	−1.032	−0.552	0.480	—	0.056	—
*POT2*	*Glyma.06G143800*	A3SS	Potassium transporter 2-like	Induce a shorted protein	—	—	—	—	0.263	—
*ARF3*	*Glyma.13G174000*	A3SS	Auxin response factor 3-like isoform	Induce a PTC	—	0.310	0.381	−0.151	−0.186	—
*ARF6*	*Glyma.15G091000*	A3SS	Auxin response factor 6-like isoform	Induce a shorted protein	−0.395	−0.370	—	−0.090	−0.077	—
*ROPGAP7*	*Glyma.02G279300*	A3SS	Rho GTPase-activating protein 7-like isoform X1	Induce a shorted protein	—	—	0.260	—	—	−0.079
*TGA21*	*Glyma.13G316900*	A5SS	Transcription factor TGA2	3′UTR, Not affected	−0.236	—	0.362	−0.100	−0.119	—
*GBF4*	*Glyma.10G223800*	A5SS	G-box-binding factor 4-like	Induce a shorted protein	—	—	—	0.065	—	—
*TGA7*	*Glyma.14G167000*	A5SS	Transcription factor TGA7-like	5′UTR, Not affected	—	0.344	—	—	−0.337	—
*GBF4*	*Glyma.10G223800*	SE	G-box-binding factor 4-like	Induce a shorted protein	—	—	—	0.080	—	—
*ETR1*	*Glyma.09G002600*	SE	Ethylene receptor isoform	Induce a shorted protein	−0.335	−0.189	—	−0.101	—	0.090

**Figure 5:**
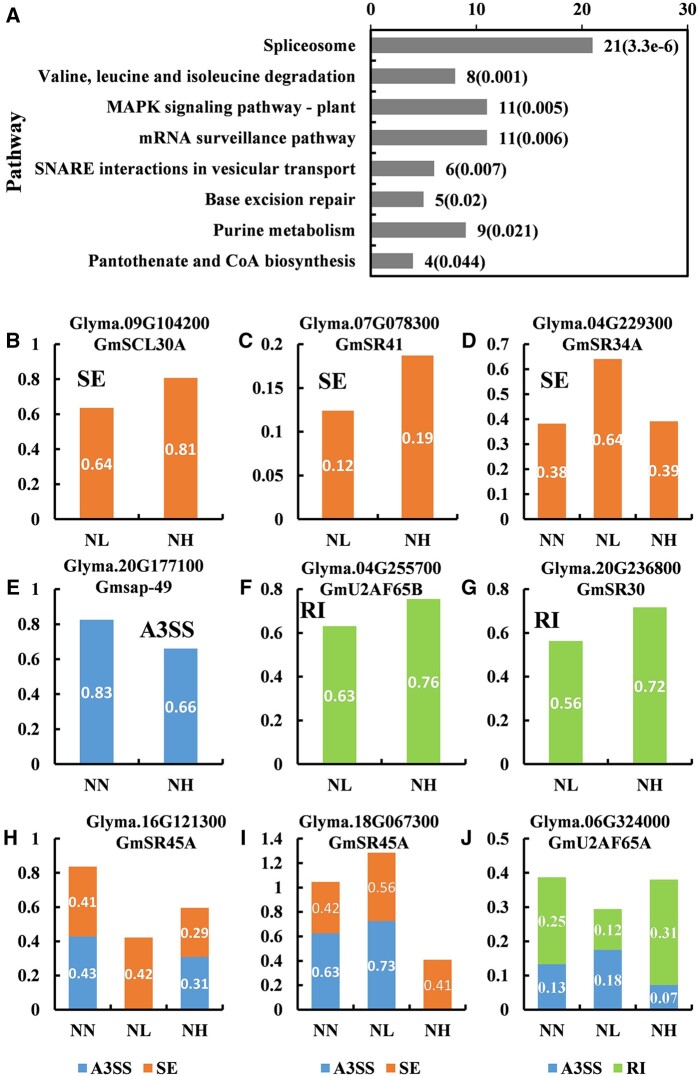
KEGG pathways significantly enriched in the DSGs, and the splicing patterns of splicing-related genes in response to nitrate treatments. (A) The KEGG pathways significantly enriched in the DSGs (*P*-value < 0.05) and the number of genes annotated in each pathway. (B–J) The relative expression levels of alternatively spliced isoforms of splicing-related genes.

We further clustered the DSGs using Kyoto Encyclopedia of Genes and Genomes (KEGG) pathway enrichment analyses. The results indicated that the spliceosome pathway was significantly enriched (*P*-value < 0.0005; Figure 5A). In addition, base excision repair pathway and the mRNA surveillance pathway were highly enriched in the DSGs (*P*-value < 0.05). To further reveal how nitrate regulates the AS patterns of splicing-related factors in the soybean root, the splicing patterns of several genes involved in those pathways were shown (Figure 5, B–J). For example, the IncLevel of splicing isoform of *GmSCL30A*, *GmSR41*, and *GmSR30* were significantly downregulated by low-nitrate treatment (Figure 5, B, C, and G). Conversely, the IncLevel of splicing isoform of *GmSR34a* were upregulated under NL condition compared with both NN and NH conditions (Figure 5D). We further investigated the expression patterns of the genes associated with the mRNA surveillance, base excision repair and spliceosome pathways, and the results indicated that the expression level of these genes were not significantly regulated by nitrate treatment, suggesting AS may act as the only regulation mode through producing different functional AS isoforms during nitrate response (Supplementary Table S2).

### Complexity and diversity of AS regulation

**Figure 6:**
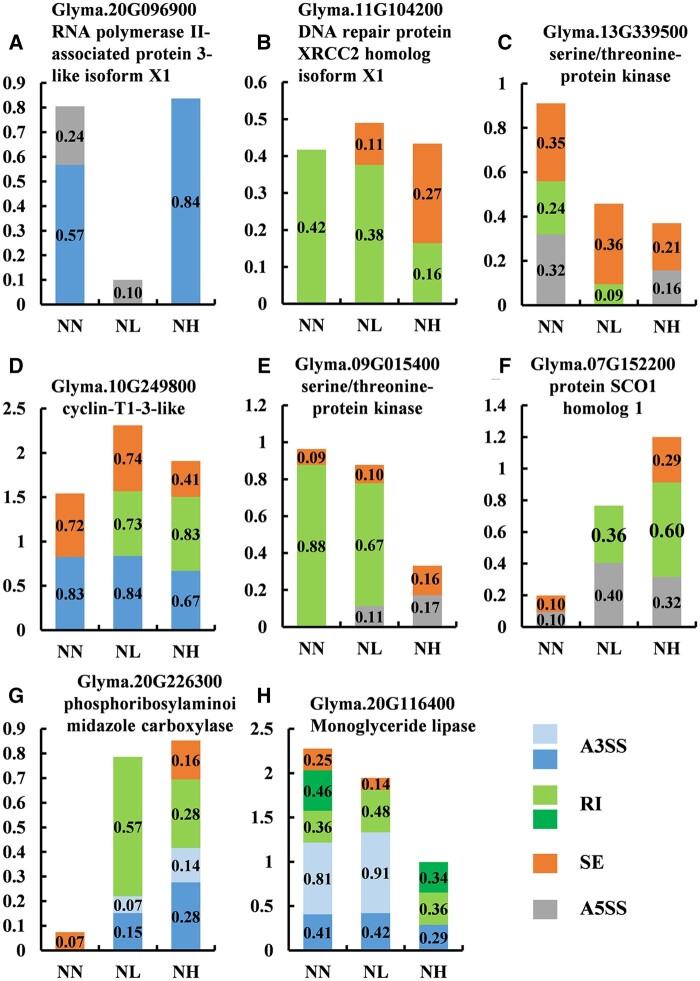
AS profiles of significantly nitrate-responsive genes under various nitrate conditions. Bars show relative expression levels of alternatively spliced isoforms of AS genes, as determined by RNA-seq. The numbers in each bar indicate the relative abundance of each isoform.

In order to illustrate the changes of AS regulation in soybean root response to nitrate, we analyzed the splicing modes of eight genes. As shown in Figure 6, we found several AS types occurred in one gene. For example, there are two and three AS types in Glyma.20G096900 and Glyma.10G249800 genes changed significantly, respectively (Figure 6, A and D). Moreover, one type of AS regulation may correspond to two different transcripts. For example, two A3SS-type events were found in Glyma.20G226300 and Glyma.20G116400 genes changed significantly, respectively (Figure 6, G and H). Two RI-type events were found in Glyma.20G116400 gene under three different nitrate concentrations (Figure 6H). Therefore, these results indicated that different AS types to be differentially regulated under different nitrate concentrations and those genes undergo a very complex and diversity regulation.

### Validation of AS and expression patterns with RT-PCR

To test how different transcript isoforms responded to nitrate levels, we investigated the expression patterns of three genes associated with AS events (Supplementary Figure S5, A–C). The expression of *Glyma.18G273100* (malectin/receptor-like protein kinase) isoform 1 was slightly induced by NL treatment, but reduced by NH treatment (Supplementary Figure S5A). Conversely, isoform 1 of *Glyma.19G147600* (unknown gene) was upregulated under NL and NH conditions (Supplementary Figure S5B). In addition, the expression levels of *Gm*.*06G324000* isoform 2 were significantly greater than those of *Gm.06G324000* isoform 1, and the expression levels of *Gm.06G324000* isoform 1 decreased as nitrate supply increased (Supplementary Figure S5C). These results suggested that nitrate concentration might have different effects on the expression patterns of different isoforms of the same gene.

To validate the nitrate-responsive AS events identified by RNA-seq, we further examined the predicated AS patterns using RT-PCR and visualized these patterns using the Integrative Genomics Viewer (IGV) (Robinson *et al.* 2017). Both isoforms were amplified in all treatment groups, but relative abundances of the isoforms differed (Supplementary Figure S5D). The relative abundance of each isoform was consistent with the RNA-seq data.

## Discussion

### AS modulations and transcriptional modulations cooperate to fine-tunes nitrate responses in soybean root

During the early seedling stage, the soybean rhizobium has not begun to fix nitrogen. Thus, the absorption of external nitrate is critical for the development of the soybean seedling (Saito *et al.* 2014). Proteins in the nitrate and peptide transporters family (NPF), which play an important role in nitrate uptake and transport, have been widely identified in plants (Longo *et al.* 2018). A previous study showed that several rice NPF genes were alternatively spliced (Huang *et al.* 2018; 2019). For example, each of *OsNPF7.7*, *OsNPF7.1*, and *OsNPF7.4* had two splicing variants in rice, and the altered expression of each variant regulated shoot branching, nirogen utilization efficiency, rice architecture, and grain yield (Huang *et al.* 2018, 2019). Recently, an analysis of AS profiles in maize revealed that over 1000 genes experienced AS regulation in response to nitrogen fluctuation (Wang *et al.* 2020). In this study, 18 NPF genes were significantly differentially expressed (Supplementary Table S3), and we found 10 NPF genes to have multiple transcripts, corresponding to a total of 15 AS events (Supplementary Table S4). In one of these genes, *NPF6.3* (*Glyma.11G031500*), the IncLevel of splicing isoform of A3SS-type AS events differed significantly between optimal- and high-nitrate conditions (Table 1). The changed transcriptional level and splicing regulation in the NPF gene family may affect soybeans to adapt to dynamic changes of nitrate content.

### Nearly 1000 nitrate-responsive AS events were identified in soybean roots under different nitrate conditions

Although AS has been studied in other plants and has been implicated in the regulation of plant nutrient responses (Dong *et al.* 2018; Huang *et al.* 2018, 2019; Wang *et al.* 2020), nitrate-response AS events have not previously been systematically analyzed and reported at the whole transcriptome level in the soybean. Here, we identified based on whole-transcriptome RNA-seq data, 335, 355, and 588 significantly nitrate-responsive AS events in soybean roots under NL, NH, and NLH conditions, respectively. The number of nitrate-responsive AS events differed substantially among the nitrate treatment groups. More nitrate-responsive AS events occurred under the NLH treatment than under the NL and NH treatments, indicating that the nitrate-responsive AS events were increasingly likely as nitrate concentration rose. Furthermore, the distributions of nitrate-responsive AS types differed at different nitrate concentrations. For example, RI-type AS events were less common in the NH treatment group compared with the NL and NLH treatment groups. Therefore, our findings not only showed that many AS events in soybean roots responded to nitrate level but also indicated that nitrate level may regulate the relative frequencies of AS event types.

### RI and A3SS were the most common nitrate-responsive AS types in soybean roots

A previous study reported that A3SS, IR, and A5SS were three primary AS events in germinating barley embryos (Zhang et al. 2016). In addition, RI was the most abundant AS type across different developmental stages in soybeans (Shen *et al.* 2014; Wang *et al.* 2014; Iñiguez *et al.* 2017), as well as the most common AS type generated in response to drought, heat, and drought plus heat in wheat (Liu *et al.* 2018). Recently, RI events were found to be the most common type of AS event during early N-responses in maize, accounting for more than one-third of all AS events based on AS profiles (Wang *et al.* 2020). In this study, although all five AS types were identified, RI and A3SS were the most common AS types, representing 67% of the all AS events across all nitrate conditions tested (Figure 1). Among the AS types that exhibited significant nitrate-response patterns, however, RI-type AS events accounted for 46.3% and 43% of all NL- and NLH-responsive AS events, respectively, more than A3SS-type AS events (23% and 24.7%, respectively; Figure 2). Therefore, the relative frequency of each AS type appeared to vary as a function of nitrate concentration. Further investigations are needed to explore these relationships.

### Go terms and KEGG pathways enriched in the DSGs

It has been shown that the kinase activity of MAPK cascade pathway related genes was regulated by AS (Castells *et al.* 2006; Koo *et al.* 2007; Lin *et al.* 2010). Here, we found MAPK signaling pathway to be significantly enriched in the DSGs (Figure 5A). It was reported that a great number of splicing factors, such as SR proteins, glycine-rich proteins), and calcium-binding proteins, are extensively alternatively spliced, and these splicing patterns may change in response to various environmental stresses (Palusa *et al.* 2007; Tanabe *et al.* 2007; Filichkin *et al.* 2010; Ding *et al.* 2014). Therefore, we expected the enrichment of the mRNA surveillance pathway and spliceosome pathway in the DSGs (Figure 5). In addition, base excision repair related genes are known to play a crucial role in repair of a variety of DNA lesions (Nimeth *et al.* 2020). Notably, several genes encoding base excision repair proteins were significantly enriched in the DSGs (Figure 5). Therefore, our results suggested that the AS of base excision repair was affected by nitrate supply. Our findings also provided new insights into the regulation of the nitrate response in the soybean root. The linkage between the AS of splicing-related factors and nitrate response in soybean root; however, requires further exploration.

A previous study showed that the interactions between nitrate and auxin played important roles in root development (Asim *et al.* 2020). In this study, the GO biological process terms most enriched in the DSGs were associated with metabolic process and cellular process (Supplementary Table S1). In particular, several auxin-responsive genes were significantly alternative spliced (Table 1). Therefore, in addition to nitrate-induced changes in gene expression levels, nitrate-induced AS events also may play vital roles in soybean root development. Primary root length, total root length, root surface area, and root volume were significantly lower under the high nitrate condition compared with no nitrate condition (Tabassum *et al.* 2021). The involvement of genes associated with the auxin signaling pathways in the nitrate response during root development is one of our future research foci. In future studies, we aim to identify key genes and regulatory pathways mediating soybean root development through the AS regulation.

## Conclusions

In summary, we identify the dynamic AS response in response to nitrate supply of soybean root and our study demonstrated that the soybean root transcriptome was regulated both at the transcript-expression level and at the alternative-splicing level. In addition, we identified molecular connections between AS and the nitrate response. Systemic approaches are needed to further disentangle and characterize soybean responses to nitrate supply to develop soybean cultivars that efficiently use high levels of environmental nitrate.
